# Anatomical Variations in the Superior Thyroid Artery: A Systematic Review and Implications for Free Flap Surgery

**DOI:** 10.3390/jcm14176250

**Published:** 2025-09-05

**Authors:** Królikowska Aleksandra, Julia Stokłosa, Alicja Patkowska, Wiktoria Rudko, Mateusz Mazurek, Zygmunt Domagała

**Affiliations:** 1 Clinical and Dissecting Anatomy Students’ Scientific Club, Medical Faculty, Wroclaw Medical University, 50-368 Wroclaw, Dolnoslaskie, Poland; julia.stoklosa@student.umw.edu.pl (J.S.); alicja.patkowska@student.umw.edu.pl (A.P.); wiktoria.rudko@student.umw.edu.pl (W.R.); mateusz.mazurek@student.umw.edu.pl (M.M.); 2 Division of Anatomy, Department of Human Morphology and Embryology, Wroclaw Medical University, 50-368 Wroclaw, Dolnoslaskie, Poland; zygmunt.domagala@umw.edu.pl

**Keywords:** superior thyroid artery, free flap surgery, thyroid gland, head and neck reconstruction

## Abstract

**Background:** The superior thyroid artery (STA) exhibits significant anatomical variability, which is crucial for head and neck surgical procedures, particularly free flap reconstruction. This systematic review synthesizes the current knowledge on STA origin, branching patterns, perfusion territory, and its relationship with the superior laryngeal nerve (SLN), focusing on implications for flap selection and surgical planning. **Methods:** A comprehensive search of relevant databases was conducted to identify studies reporting on STA anatomy. Data extraction focused on the STA origin variations, relationships with anatomical landmarks, branching patterns, perfusion territory, and the STA-SLN relationship. Emphasis was placed on variations impacting STA’s suitability as a recipient vessel for free flaps. Data were synthesized qualitatively. **Results:** The STA most commonly originates from the external carotid artery (ECA), with variations from the carotid bifurcation (CB) and common carotid artery (CCA). Sex-based and laterality differences were noted. Branching patterns varied considerably, influencing perfusion of the thyroid gland, larynx, and adjacent musculature. The STA’s relationship with the external branch of the SLN (EBSLN), classified by Cernea’s classification, highlighted the risk of iatrogenic injury. The STA provides perfusion to the thyroid gland, larynx, sternocleidomastoid muscle, and strap muscles, all of which can be raised as flaps. **Conclusions:** Understanding STA anatomical variations is essential for surgeons planning free flap reconstruction in the head and neck. This review underscores the importance of preoperative imaging to assess STA suitability as a recipient’s vessel and minimize complications. Further research is needed to quantify the impact of STA variations on free flap outcomes.

## 1. Introduction

Anatomical knowledge of the superior thyroid artery is paramount in head and neck surgery, particularly when planning free flap reconstruction. The STA, a major vessel supplying the thyroid gland, larynx, and surrounding structures, demonstrates considerable variability in its origin, branching patterns, and relationship with adjacent neurovascular structures.

These variations have significant clinical implications, especially in selecting the STA as a recipient vessel for free flaps. A lack of understanding of such variations can lead to an increased risk of hemorrhage, nerve injury, and flap failure. This systematic review aims to synthesize the current literature on STA anatomy, focusing on its origin, branching patterns, perfusion territory, and relationship with the SLN. The review was designed to address the following research questions:

1. What are the prevalence and types of anatomical variations in the origin, branching patterns, and perfusion territory of the STA?
2. How do these variations relate to the SLN and to relevant surgical landmarks?
3. What are the implications of these variations for surgical planning, including the selection of recipient vessels in free flap reconstruction and the prevention of iatrogenic nerve injury?

## 2. Materials and Methods

### 2.1. Study Protocol and Registration

This review adhered to a pre-designed protocol (Supplementary File S1) and followed the rigorous standards for conducting systematic reviews outlined in the Preferred Reporting Items for Systematic Reviews and Meta-Analyses (PRISMA) guidelines. Before the data extraction, this review protocol was registered in the international systematic review protocol database PROSPERO (number of registrations: CRD420251009471), ensuring the highest level of quality and reliability.

### 2.2. Search Strategy

**Figure 1:**
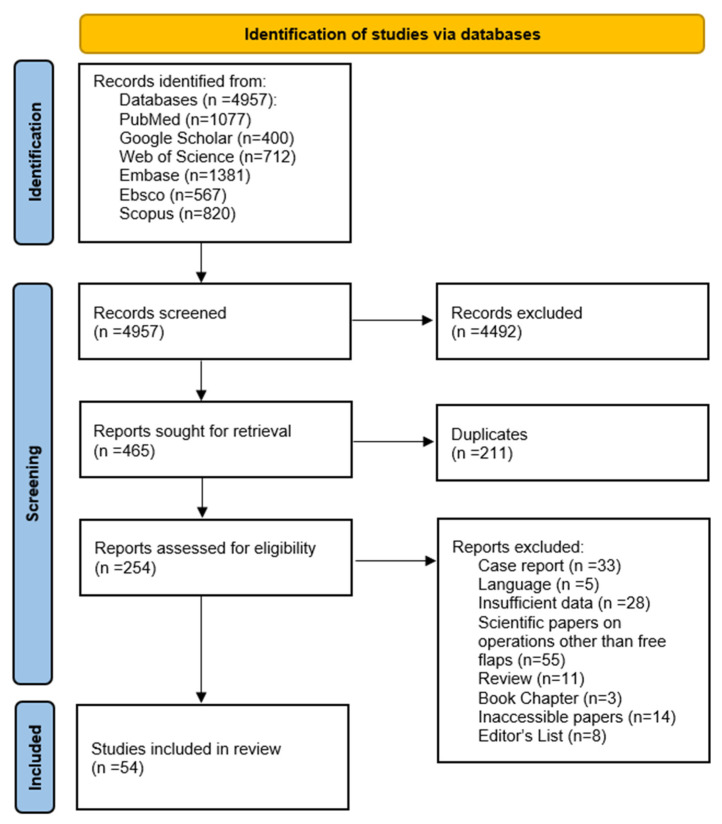
PRISMA (Preferred Reporting Items for Systematic Reviews and Meta-Analyses) flowchart.

We searched all the literature on the superior thyroid artery and its usage in free flap surgeries until January 2025. We searched PubMed, Embase, EBSCO, Web of Science, Scopus, and Google Scholar. All the results of PubMed, Embase, EBSCO, Web of Science, and Scopus Databases were revised. We screened the first 200 results on Google Scholar, which is sufficient according to the literature. The search terms we used were superior thyroid artery anatomy and superior thyroid artery surgery. We did not make any language restrictions during the search. Two authors independently screened all the articles found using abstracts, titles, and keywords. All references of the found studies were also screened; if any were deemed valid, we included them in our review. The whole search strategy is presented in Figure 1 and Supplementary File S2.

### 2.3. Selection Criteria

To be included in the systematic review, the article had to meet specific criteria, presented here in hierarchical order:

1. It had to concern the anatomy of the superior thyroid artery or the usage of the superior thyroid artery in free flap surgery.
2. It had to be a cadaveric, radiological, or surgical study.
3. It had to be written in English.
4. It had to be an original work presented as a complete, peer-reviewed article.
5. It had to clearly state its objectives, methods, and results.

Two independent reviewers screened the studies. The first screening was completed by AP, JS, and WP, the second was performed by AK. All the reviewers were familiar with the selection criteria. All were instructed to include studies for which they needed clarification. A second reviewer evaluated these studies and included or rejected them based on the discussion. If a consensus was not reached, the fifth reviewer, MM, was consulted to solve the problem.

### 2.4. Study Appraisal

To decrease the risk of bias in our systematic review, we used study appraisal tools based on the current consensus on systematic review methodology. To assess the methodological quality, we used an AQUA Checklist. This tool consists of Title, Abstract, Introduction, Methodology, Results, Discussion, Conclusions, Acknowledgement, Conflict of Interest, and Funding. During the AQUA analysis for each paper, the authors paid particular attention to the limitations of the study presented by the authors, the conflicts of interest presented by the authors and the authors’ use of checklists and tools to prevent errors. All the scores obtained by the studies included in our review are presented in Table 1.

### 2.5. Data Extraction

The data was extracted using the data extraction form created by AK according to the Cochrane Handbook of Systematic Reviews of Interventions, chapter 5.3. After AP, JS, and WR extracted the data, AK re-screened it.

The primary data we collected were the first author’s surname, publication year, and the type of study. More precise data we searched for included information on the aim of the study, methodology, population group, and results. Any discrepancies were dissolved by discussion between the team members until a conclusion was reached. The data are presented in an extraction table, graphs, tables, and figures for each chapter.

### 2.6. Outcome Analysis

Outcomes were analyzed qualitatively, as the study primarily synthesizes the existing literature on the anatomical variations in the STA and their implications for free flap surgery. The review focuses on summarizing the frequency of STA origin variations, branching patterns, perfusion territories, and relationships with adjacent structures, such as the SLN. Quantitative data, where available, were reproduced from individual studies, but the methodological variability across studies precluded a formal meta-analysis. The article is structured into four main sections: introduction, materials and methods, results, and conclusions. In Section 3, the authors discuss the anatomical variations in the STA, including its origin, branching patterns, and relationship with the SLN, and summarize the implications of these variations for surgical planning and free flap reconstruction. The conclusions emphasize the importance of preoperative imaging and suggest future research directions to quantify the impact of STA variations on surgical outcomes.

No methods were used to assess the certainty (or confidence) in the body of evidence for any outcome, as a formal synthesis of the results across studies was not performed.

## 3. Results

### 3.1. Origin of STA

Sreedharan et al. reported that the STA most commonly originates from the ECA (88.33%), less frequently from the CB (8.33%), and rarely from CCA (3.33%) [47]. Similar results were obtained by Sharma et al., who studied thirty right STAs, finding that 90% arose from the ECA, while only two originated from the CB and one from the CCA [32] (Table 2).

Patel et al. analyzed the origin of the STA concerning the carotid bifurcation, observing that, in most cases, the STA originated above the bifurcation (77%) and less frequently at the same level of the bifurcation (23%). No cases of STA originating below the bifurcation were noted [12]. Cobiella et al. classified STA origins relative to other anterior branches of the ECA, identifying four patterns: separate origins of all branches (80.83%), a common trunk for lingual and facial arteries with an independent STA (17.62%), a shared trunk for STA and lingual artery with a separate facial artery (1.04%), and a common trunk for all three (0.52%) [3].

#### 3.1.1. Influence of Sex and Laterality

Several studies have examined the influence of sex on STA origin. Sharma et al. demonstrated that, in male cadavers, 90.90% of right STAs originated from the ECA, while in female cadavers, the proportion was 87.50%, suggesting a slightly higher prevalence in males [32]. Bunea et al. conducted a study using 134 CT scans and found similar results, with 86.49% of female STAs and 88.33% of male STAs arising from the ECA [20]. Herrera-Núñez et al. identified significant sex differences in STA origin, reporting that the STA arose from the ECA in 50% of females and 59% of males, from the CCA in 16% of females and 12% of males, and from the CB in 9% of females and 28% of males [44].

Laterality differences were also noted. Herrera-Núñez et al. observed that the right STA originated from the ECA in 63.2% of cases compared to 48.7% on the left. Conversely, the left STA arose from the CCA (26.3%) and CB (25%) more frequently than the right STA (10.5% and 23.7%, respectively) [44]. Shyamala et al. analyzed 60 STAs (33 right and 27 left) and found that variations were more frequent on the left side. The left STA originated from the CB in 15 cases and from the CCA in 3 cases, whereas the right STA arose from the CB in 13 cases and never from the CCA. The study further demonstrated that the STA more frequently originated from the ECA in males (51%) than in females (37%), whereas in females, STA origin from the CB (58%) was more prevalent than in males (44%) [15] (Table 3).

#### 3.1.2. Common Variations and Additional Arterial Connections

Beyond common origin sites, certain studies have identified unusual STA origins and associations with adjacent arteries. Bunea et al. documented cases of a common STA origin with the thyrolingual trunk (three cases) and the thyrolingofacial trunk (two cases) [20]. Similarly, Kapre et al. identified thyrolingual trunks in two cadavers out of twenty-one, exclusively on the right side—one originating from the CCA and the other from the CB [25]. An interesting case of common origin was also presented by Jitpun et al., who described STA that originated in a common trunk with the occipital, lingual, and facial arteries [40].

### 3.2. Relationship with Anatomical Landmarks

Several authors examined the distance between the STA origin and anatomical landmarks. Abdalla et al. found that the most frequent STA origin site was at the level of the greater horn of the hyoid (45% of cases), followed by the body of the hyoid (32.8%) and the superior border of the thyroid cartilage (18.8%) [1]. Al-Rafiah et al. reported that when the STA originated above the CB from the ECA (3.3% of cases), the distance ranged from 0.9 cm to 1.1 cm. When originating below the CB from the CCA (18.3%), the distance varied between 0.4 cm and 1.0 cm. The majority (76.7%) of STAs stemmed from the CB, with one case (1.7%) arising from a thyrolingofacial trunk [14]. Additionally, in 98.3% of cases, the STA arose from the anteromedial surface of the ECA, while in 1.7%, it originated from the lateral surface, running anteriorly to the CCA before descending [14].

Lucev et al. found that when the STA originated from the ECA, its distance from the CB ranged from 2 mm to 10.5 mm, whereas from the CCA, the distance varied between 2 mm and 10.7 mm [19]. Sasikumar et al. analyzed gender differences and found that in males, the distance between STA origin and CB was 4.5 ± 1.6 mm on the right and 4.22 ± 2.8 mm on the left. In females, the distances were 5.42 ± 3.5 mm on the right and 4.8 ± 2.1 mm on the left [46]. Griepp et al. used the medial border of the clavicle as a reference point and found the mean STA origin distance to be 105.18 mm [41].

### 3.3. Correlation with Carotid Bifurcation Levels

Esen et al. classified carotid bifurcation levels according to cervical vertebrae, finding that patients with CB at lower cervical levels (C5–C7) had a higher likelihood of STA arising from the ECA, while those with higher CB (C1–C3) more commonly had STA originating from the CCA [31]. Ongeti et al. corroborated this finding, observing that all cadavers with STA arising from the CCA had high CB levels [36]. Lo analyzed the correlation between STA origin and CB level using anatomical landmarks, demonstrating that when the CB was at the greater tip of the hyoid, 88.9% of STAs originated from the CB and 11.1% from the ECA [7]. When the CB was at the superior thyroid cartilage level, 42.3% arose from the CB and 57.7% from the ECA [7].

The superior thyroid artery typically gives rise to multiple branches supplying the thyroid gland, larynx, and adjacent musculature. The most commonly observed branches include the infrahyoid branch, which courses along the hyoid bone; the sternocleidomastoid branch, supplying the sternocleidomastoid muscle; the cricothyroid branch, which runs towards the larynx; the superior laryngeal artery (SLA), a clinically significant branch closely associated with the superior laryngeal nerve; and the glandular branches, which further divide into anterior and posterior subdivisions to vascularize the thyroid gland. While this typical branching pattern is frequently encountered, anatomical variations in these branches’ number, origin, and course have been widely documented.

Al-Azzawi et al. reported a variation in which only four branches arose from the STA, omitting one of the typically described divisions. In this configuration, the infrahyoid branch was the most superior, following the hyoid bone. The sternocleidomastoid branch emerged laterally from the STA and descended toward its target muscle, while the cricothyroid branch coursed toward the larynx. The SLA, a branch of notable surgical importance, traveled toward the thyroid gland and maintained proximity to the superior laryngeal nerve throughout its course [2].

**Figure 2:**
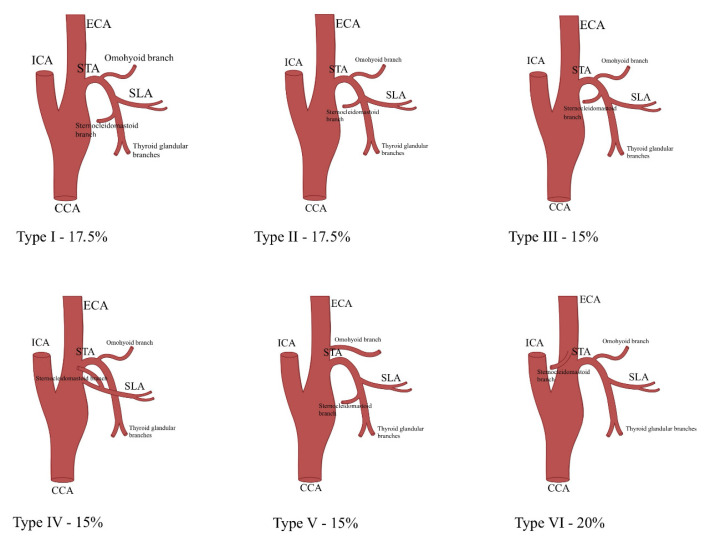
Branching patterns of the superior thyroid artery [11]. Abbreviations: CCA—common carotid artery; ECA—external carotid artery; ICA—internal carotid artery; STA—superior thyroid artery; SLA—superior laryngeal artery.

Ozgur et al. categorized STA branching patterns into six types. In type I (17.5%), the SLA and infrahyoid branches separated first, with the sternocleidomastoid and thyroid glandular branches diverging below. Type II (17.5%) showed an independent infrahyoid branch, while SLA, thyroid glandular, and sternocleidomastoid branches originated from a common trunk. Type III (15%) featured consecutive branching: infrahyoid, sternocleidomastoid, SLA, and thyroid glandular branches. In type IV (15%), the SLA arose from the ECA, with the sternocleidomastoid artery branching from it. Type V (15%) showed the SLA arising first, followed by the sternocleidomastoid and thyroid glandular arteries, while the infrahyoid branch originated from the ECA. Type VI (20%) featured an infrahyoid branch arising first, then the SLA, followed by the thyroid glandular branch, with the sternocleidomastoid artery stemming from the ECA [11] (Figure 2).

Ozgur et al. further analyzed individual branch origins, noting that the infrahyoid branch originated from the STA in 85% of cases and from the ECA in 15%, descending along the hyoid bone and piercing the thyroid membrane. The SLA consistently traveled along the internal branch of the superior laryngeal nerve. The sternocleidomastoid artery arose from the STA in 80% of cases and from the ECA in 20%, traversing laterally across the carotid sheath. The cricothyroid artery originated from the anterior glandular branch in 70% of cases and from the STA in 30%, crossing the anterior cricothyroid ligament and anastomosing with its contralateral counterpart. The anterior glandular branch typically crosses the isthmus, forming anastomoses with anterior and posterior glandular branches. The posterior glandular branch supplied the posterior thyroid border [11].

Other studies reported varied results. Anand et al. used Ozgur’s classification but found different prevalence rates, with type I in 56.25% of cases, type II in 38.75%, and type III in 5%. This study also noted that the infrahyoid and sternocleidomastoid branches originated exclusively from the STA, while the cricothyroid artery consistently arose from the anterior glandular branch [18]. Devadas et al. found that the SLA stemmed from the STA in 91.7% of cases, whereas Rusu et al. reported 68% of SLA originating from the STA and 32% from the ECA [27,34].

Gupta et al. examined the correlation between STA origin and branching patterns. Type I (28%) described STA arising from the ECA and bifurcating into two major branches. Type II (20%) featured a trifurcation of STA from the ECA, while type III (52%) represented STA originating from the CB without bifurcation or trifurcation. An unusual case of STA stemming from the internal carotid artery was also noted [26].

### 3.4. Superior Thyroid Artery Perfusion Territory

A total of nine studies investigated the perfusion territory of the superior thyroid artery, which includes the larynx, thyroid gland, neck muscles, and overlying skin [26]. The thyroid gland receives its blood supply from the anterior and posterior glandular branches of the STA [18].

Additionally, the STA contributes to vascularizing the parathyroid glands through anastomoses with the inferior thyroid artery (ITA) [39]. These anastomoses occur in 24.9% of superior parathyroid glands and 6.8% of inferior parathyroid glands, while in 8.5% of cases, the STA serves as the sole blood supplier to the parathyroid glands. Furthermore, 61% of superior parathyroid glands exhibited asymmetric vascularization, receiving blood from different vessels bilaterally. Similarly, the inferior parathyroid glands showed no significant symmetry, with 34% not being supplied by the same blood vessel on both sides [39] (Table 4).

The STA also plays a crucial role in supplying the sternocleidomastoid muscle (SCM). Kierner et al. demonstrated that the STA and the ECA almost equally contribute to the vascularization of the middle third of the SCM, with the STA being the sole supplier in approximately two-thirds of cases [23]. Hu et al. further reported that the STA and occipital arteries are the primary sources of blood supply to the SCM among the ECA branches. The STA branch supplying the SCM enters the middle portion and provides blood to the lower half of the muscle [6] (Table 5).

**Figure 3:**
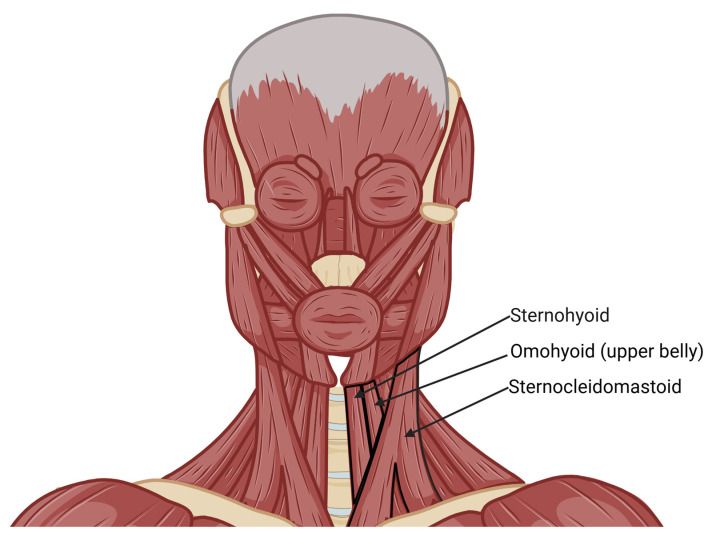
Parts of muscles supplied by superior thyroid artery [23,29].

The STA also perfuses strap muscles. Wang et al. documented that an STA branch primarily supplies the upper part of the SH muscle, the thyrohyoid (TH) muscle, and the upper belly of the omohyoid (OMO) muscle. The SH muscle receives its blood supply directly from the STA in 25% of cases, whereas in 75%, it originates from the STA’s cricothyroid artery. Similarly, the TH muscle is supplied by the cricothyroid artery or directly by the STA in 75% and 13% of cases, respectively. The OMO muscle receives its blood supply from STA branches in 75% of specimens, with origins from either the main trunk (31%) or the cricothyroid artery [29] (Figure 3).

The cutaneous blood supply of the neck has also been examined. Rabson et al. conducted a cadaveric study that revealed STA branches supplying the platysmal vascular plexus and the overlying dermal–subdermal plexus [35]. Additionally, the STA contributes to the tracheal blood supply, as demonstrated by Salmeron et al., who perfused specimens with India ink. Staining was observed in the epiglottis, larynx, and trachea, emphasizing the importance of STA-ITA anastomoses. Mucosal staining was present on an average of three tracheal rings, with maximal staining reaching the fifth tracheal ring [33].

The STA is also significant in supplying the external branch of the superior laryngeal nerve (EBSLN), which innervates the cricothyroid and inferior pharyngeal muscles. Yalcin et al. reported that, in 50% of cases, the EBSLN received its blood supply from a branch of the posterior glandular branch of the STA, while in 44.23%, it originated from the anterior glandular branch. In the remaining cases, the branch to the EBSLN arose from the main STA trunk, the infrahyoid branch, or the bifurcation of STA at the level of the glandular branches [17].

### 3.5. The Relationship Between the Superior Thyroid Artery and the Superior Laryngeal Nerve

The anatomical relationship between the STA and the SLN is of critical importance in neck surgery, particularly during thyroid and laryngeal procedures. Due to its proximity to the STA, the EBSLN is at risk of iatrogenic injury, which can lead to voice alterations and impaired laryngeal function. Understanding the variations in their spatial arrangement is essential for surgeons to minimize complications and preserve nerve integrity.

**Figure 4:**
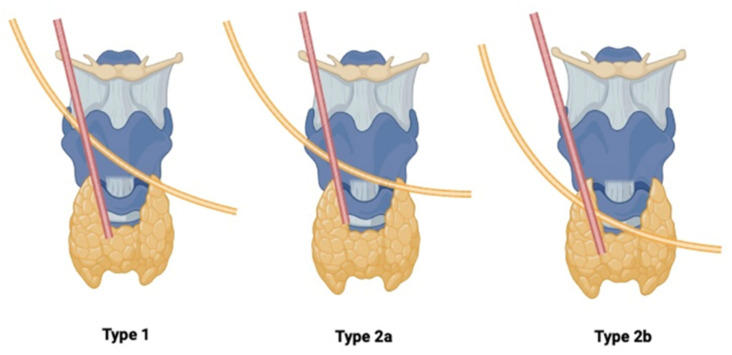
Cernea’s classification [28].

Dessie et al., in a study of 43 embalmed cadavers, analyzed the STA-EBSLN relationship using Cernea’s classification: type 1, type 2a, and type 2b. In type 1 (57%), the EBSLN crosses the STA at a distance of ≥1 cm above the upper thyroid pole. In type 2a (40.7%), the crossing occurs at <1 cm above the pole, while in type 2b (2.3%), the EBSLN crosses below the thyroid pole [28] (Figure 4).

Ahmad et al., in an intraoperative study of 50 patients, found type 1 in 53.2%, type 2a in 17.7%, and type 2b in 22.5%, with 6.4% showing no specific pattern [38]. In contrast, Devaraja et al., examining 10 cadavers, reported type 2a as the most common, differing from previous findings, likely due to the smaller sample size [30] (Table 6).

Estrela et al. conducted a cadaveric study measuring distances between the EBSLN, STA, and thyroid gland. The mean distance between the EBSLN and superior thyroid pole was 7.68 ± 3.07 mm, and the tangent from the inferior thyroid cartilage edge to the EBSLN-STA intersection measured 4.24 ± 2.67 mm. The distance between the EBSLN-STA intersection and the superior thyroid pole was 9.53 ± 4.65 mm, while the EBSLN–centerline distance to the posterior thyroid cartilage point was 19.70 ± 2.82 mm [4].

Lu et al. classified ELN loops in 76 specimens into three types: type I (>1 cm above the superior thyroid pole, 55 cases), type II (<1 cm above, 16 cases), and type III (below the pole, 5 cases). Type I loops were distant from the thyroid pole, making them less relevant in surgical implications. Types II and III were further categorized based on their relationship with STA branches [8].

**Figure 5:**
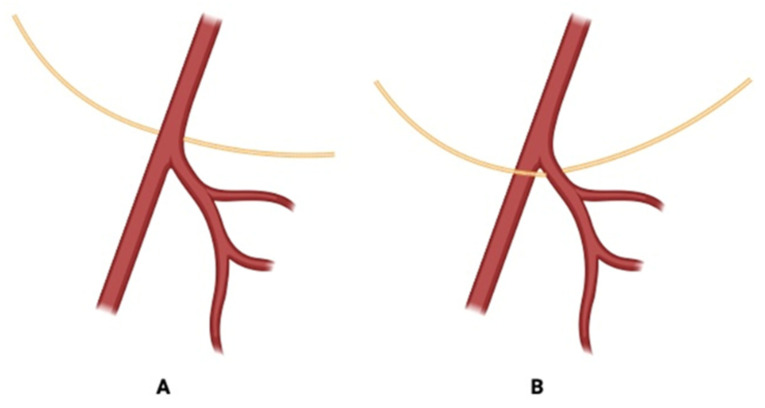
Relationship between the STA, its branches, and EBSLN. (**A**)—nerve medial to the artery 71.9%; (**B**)—nerve in between the branches of the artery 28.1% [13].

Manjappa et al. measured the distance from the superior thyroid pole to the point where the ELN turned medially to STA. The distance was >10 mm in 20 cases, <10 mm in 10 cases, and exactly 10 mm in 10 cases [9]. Poyraz et al. found that among 32 dissected EBSLNs (16 on each side), 71.9% were medial to STA, and 28.1% were between STA branches, with none lateral to STA [13] (Figure 5).

Yalcin et al. analyzed ELN positions relative to STA: medial (76.5%), lateral (20.9%), and posterior (2.4%) [16]. Haller et al. investigated the STA-ESLN relationship, noted on which level of the vertebral column it was located, and compared it to ISLN. Many varieties of ESLN were found, but they were always a safe distance above the C3–C4 and below C6–C7 [5]. Magoma et al. examined ELN-STA relationships in an African population, finding that 25% of ELNs crossed the STA within 1 cm above the upper thyroid pole, and the remaining 75% of ELNs crossed the artery more than 1 cm above the pole. Bilateral symmetry was noted in all cases [42]. Kierner et al. classified four types of EBSLN-STA relationships. In type 1, the EBSLN crossed the STA more than 1 cm above the upper pole of the thyroid gland. In type 2, the EBSLN crossed the STA less than 1 cm above the upper pole of the thyroid gland. In type 3, the EBSLN crossed the STA under cover of the upper pole of the thyroid gland, while in type 4 the EBSLN descended dorsally to the artery and crossed the branches of the STA immediately above the upper pole of the thyroid gland [24]. Monfared et al. described the relationship between SLN and its external and internal branches with STA in the context of the microsurgical anatomy of laryngeal nerves related to thyroid surgeries. The IBSLN passed close to the STA and descended towards the thyrohyoid membrane, whereas the EBSLN traveled deeper, parallel to the STA and toward the cricothyroid muscle. It was also noted that the branches of STA supplied the branches of SLN: the superior laryngeal artery supplied the IBSLN, and cricothyroid artery supplies the EBSLN [43].

### 3.6. Landmarks to Use During Surgeries

**Figure 6:**
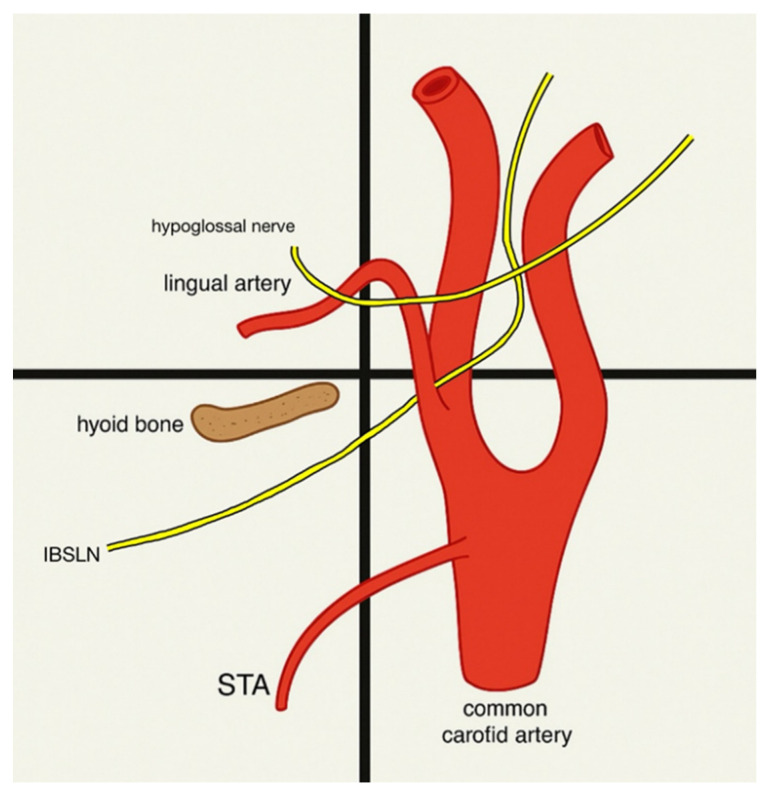
The significance of the tip of the greater horn of the hyoid bone (THB) in the localization of STA and SLN [21].

A total of four studies have investigated landmarks connected to STA and SLN or STA and SLN for use in surgeries conducted in the neck region. Lemaire et al. conducted a study assessing the relevance of the tip of the greater horn of the hyoid bone (THB) in the localization of STA and SLN. Thirty perfusion-fixed human cadavers were used in this study, with sixty preparations on each side. There was no significant difference between the averages for each side. THB was used as a landmark when finding STA and SLN. The distances of those structures from THB were measured, and the mean and standard deviation for each measurement were calculated. The origin of STA lay 13 ± 4.5 mm below and 4.7 ± 2.7 mm behind the THB. In each specimen, SLN divided into IBSLN and EBSLN at the level of the hyoid bone [21] (Figure 6).

Lee et al. researched whether the STA is in the safety zone during the percutaneous cervical approach at different levels. At C3–C4, the safety zone contained STA in 86.7% of cases; at C4–C5, STA was located in the safety zone in 26.7% of cases. At C5–C6 and C6–C7, the STA was not located in the safety zone, so STA can only be used as a safety landmark at C3–C4 and C4–C5 [22]. Park et al. present a study that shows the possibility of using SLN and its branches, ISLN and ESLN, and STA as landmarks in the C2–C3 level for the anterior cervical approach (ACA). The SLN descends into the ACA field, dividing into the ESLN and ISLN. The levels for the ISLN were C3 with 52.8% frequency, C2 with 19.4% frequency, and C3–C4 with 11.1% frequency. The levels for the ESLN were C2–C3 with 13.9% frequency, C3 with 33.3% frequency, and C3–C4 with 22.2% frequency. The levels for STA were C3 with 44.4% frequency and C3–C4 with 22.2% frequency [37]. Ortega et al. present the landmarks that can be used to find a branch of SLN, the ELN, during surgeries. In 100% of 157 embalmed human cadavers, the ELN was located deep in the ascending pharyngeal vein. ELN crosses the carotid axis at the origin of the STA in 47% of cases. ELN passes medial to origin of STA in 89% cases. It travels through the inferior pharyngeal constrictor in 80% of cases. The STA and ascending pharyngeal vein are most reliable for use as landmarks when finding ELN [45] (Table 7).

### 3.7. The Role of the Superior Thyroid Artery in Free Flap Surgery

A retrospective study by Nakamura et al. investigated postoperative thyroid function in 91 patients who underwent free jejunal transfer after total pharyngolaryngoesophagectomy with hemithyroidectomy. Patients were stratified into two groups based on recipient vessel selection: one in which the contralateral STA was utilized, and another in which alternative arteries were used, thus preserving the STA. The authors reported no significant differences in the incidence of postoperative hypothyroidism or subclinical hypothyroidism between groups, concluding that the STA can be safely used even in patients with a single remaining thyroid lobe, provided that the inferior thyroid arteries are preserved. By contrast, the findings of Pak et al. present a different perspective. In a cohort of 101 reconstructive cases, 40 patients underwent anastomosis utilizing the STA. This group demonstrated significantly higher mean postoperative TSH levels compared with patients in whom other recipient vessels were used (7.34 vs. 2.87 mU/L; *p* = 0.02). Moreover, 27.5% of patients in the STA group developed subclinical hypothyroidism, and 17.5% required initiation of levothyroxine supplementation. Although the observed biochemical abnormalities were largely subclinical, these findings suggest that the use of STA may predispose some patients to impaired thyroid function.

Nonetheless, the superior thyroid artery (STA) is one of the most commonly used recipient vessels in head and neck microvascular free-flap reconstruction, due to its anatomical consistency and accessibility, as well as the compatibility of its size with donor vessels. Arising from the anterior surface of the external carotid artery, the STA descends toward the thyroid gland following a relatively superficial course. Unlike the facial or lingual arteries, which are often ligated or damaged during neck dissection, the STA remains intact in most cases, making it a reliable recipient vessel for free flap surgery [53]. Its diameter, which ranges from 1.5 to 3.1 mm, is well-matched with common donor arteries such as the radial artery (2.3 mm), the lateral circumflex femoral artery (1.75–3 mm), and the peroneal artery (1–2 mm). Furthermore, its location within the carotid sheath offers additional protection against iatrogenic injury [45,53]. The STA’s anterior neck position makes it more accessible than deeper vessels, and its proximity to both the external and internal jugular veins simplifies venous anastomoses [50].

**Figure 7:**
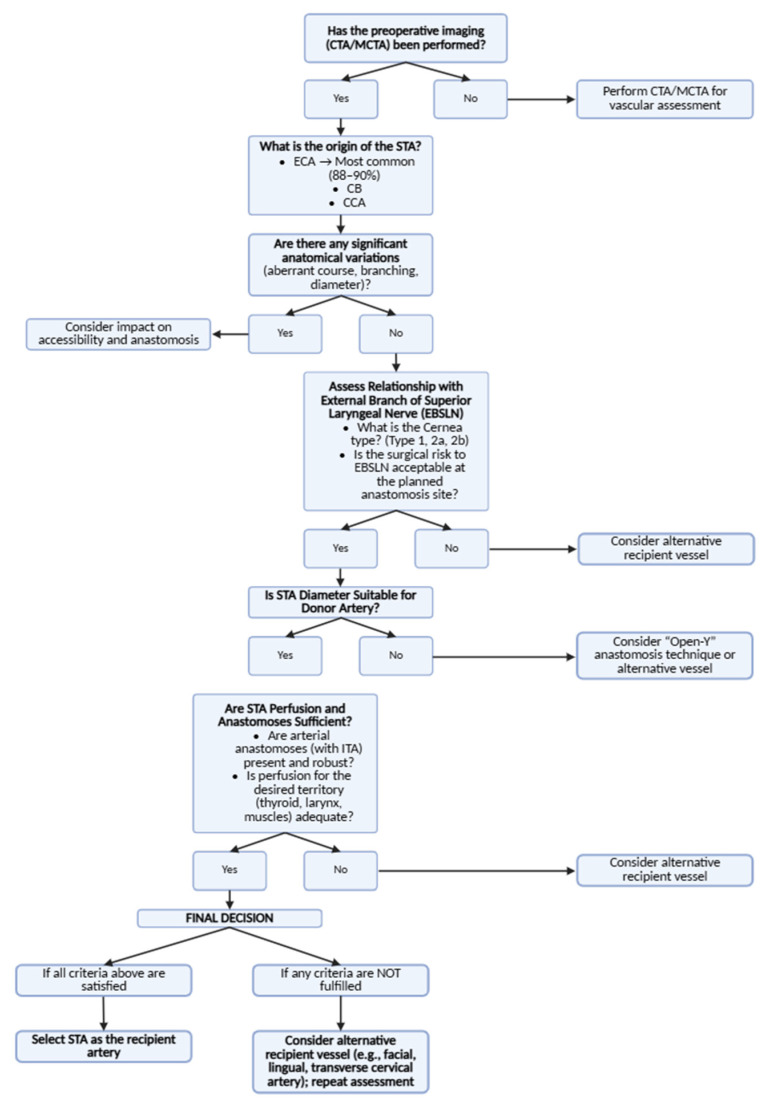
Clinical decision-making algorithm for the use of the superior thyroid artery as a recipient vessel in head and neck free flap reconstruction.

In a study evaluating 337 free flaps, the STA was successfully used as a recipient vessel, with an overall flap success rate of 98.6% [53]. However, one of the primary challenges associated with using the STA as a recipient vessel is the potential size mismatch between the donor and recipient arteries, which can increase turbulence at the anastomotic site and elevate thrombosis risk [53]. To address this, the “Open-Y” anastomosis technique was introduced [53]. This method involves creating a Y-shaped opening in the STA, allowing for a more gradual transition in the vessel diameter, reducing turbulence, and improving anastomotic stability [53]. Clinical studies have shown that the “Open-Y” technique yields a 98.6% success rate, compared to 97.4% for conventional end-to-end anastomoses, and is associated with lower complication rates, including a reduced incidence of venous thrombosis [53] (Figure 7).

When selecting recipient vessels for free flap surgery, the STA is often compared with other available options in the head and neck region, including the facial artery (FA), lingual artery (LA), and transverse cervical artery (TCA). Each of these vessels has distinct characteristics that influence their suitability for microvascular anastomosis. The FA, despite its larger caliber, is frequently sacrificed during neck dissections, limiting its utility [45]. The LA, although offering excellent perfusion, is more technically challenging to access due to its deep anatomical location [45]. The TCA is valuable in vessel-depleted cases but lacks the STA’s consistent anatomical reliability [52]. In contrast, the STA remains preserved in most cases and provides easier exposure, making it a preferred recipient artery, particularly in secondary or complex procedures.

The utility of the STA is also well-demonstrated in mandibular reconstruction using the free fibula flap (FFF), a gold-standard approach. When ipsilateral recipient vessels are unavailable, the STA serves as a primary anastomotic site [48]. A cadaveric study by Kasai et al. confirmed that the STA was reachable in 100% of cases of contralateral mandibular reconstruction, further highlighting its versatility [48].

Anatomical studies have shown that anastomoses between the STA and the ITA are present in 100% of cases. These consistent vascular connections provide perfusion redundancy, which is particularly beneficial in free flap reconstruction [50]. Chung et al. emphasized that these anastomoses provide a robust vascular network, enhancing the reliability of the STA as a recipient vessel [50]. Kasai et al. demonstrated that the STA’s consistent anatomy and accessibility make it a highly reliable option for microvascular anastomoses, even in complex reconstructive scenarios such as contralateral mandibular reconstruction [48]. This redundancy in vascular connections, combined with the STA’s anatomical consistency, further enhances its suitability as a recipient vessel in free flap surgery [48,50].

The STA has also been explored as a vascular source for perforator-based flaps, particularly the STA perforator (STAP) flap. This thin fasciocutaneous flap, nourished by perforating branches of the superior thyroid artery, has shown great potential for reconstructing small soft tissue defects in the head and neck region [54]. The STAP flap offers several advantages: it ensures reliable perfusion through the STA’s perforator branches, provides a thin and pliable tissue ideal for facial and submental reconstructions, and results in minimal donor site morbidity [48,54].

In salvage and multi-flap procedures, the STA becomes increasingly important, especially in vessel-depleted necks. When other recipient vessels such as the FA or LA have already been used, the STA often remains viable [51]. Lai et al. reported that among patients requiring three or more free flap procedures, the STA was used in 16.9% of cases [51]. This highlights its value as a “lifeline” in long-term reconstructive strategies.

## 4. Discussion

The STA demonstrates substantial anatomical variability in its origin, branching pattern, and neurovascular relationships. These differences hold significant clinical relevance, particularly in head and neck microsurgery, where the STA is frequently utilized as a recipient vessel for free flap reconstruction. The ECA remains the most common origin of the STA, although variations involving the CB and CCA are not uncommon. Such variations may influence flap design, anastomosis strategy, and ultimately, surgical outcomes.

Equally important is the relationship between the STA and the EBSLN. Given their close anatomical proximity, careful intraoperative dissection is essential to minimize the risk of vocal dysfunction due to iatrogenic nerve injury. Awareness of Cernea’s classification and preoperative imaging can improve the safety of neck procedures by guiding the dissection planes around the STA.

Beyond its established role in free flap procedures, recent innovations have expanded the STA’s surgical utility. STAP flaps represent a promising option for reconstruction of smaller defects, offering thin, well-vascularized tissue with low donor site morbidity [54]. Additionally, in complex or vessel-depleted neck reconstructions, such as contralateral fibula free flap mandibular repairs, the STA has shown consistent accessibility and reliability [48]. These advances are supported by the increasing use of computed tomography angiography (CTA), which enables accurate preoperative vessel mapping and recipient site planning [49].

The STA also serves a valuable function in vascular and endocrine surgery. It is a reliable anatomical landmark during carotid endarterectomy and aids in avoiding critical neurovascular structures [10]. In thyroid surgery, preserving the STA is crucial to maintain perfusion to the thyroid and parathyroid glands, thereby reducing the risk of postoperative hypoparathyroidism [39]. These roles underline the STA’s importance beyond reconstructive microsurgery, reinforcing the need for detailed anatomical knowledge in diverse surgical disciplines.

Future research should continue to focus on integrating preoperative imaging modalities with anatomical classification systems to optimize surgical planning. Longitudinal outcome studies evaluating STA-based anastomoses across diverse patient populations would further elucidate the clinical significance of STA variability.

This review has several methodological limitations. Firstly, no formal synthesis of results was conducted due to the heterogeneity of the included studies in terms of methodology, anatomical definitions, and reporting standards. As a result, it was not possible to perform a meta-analysis or assess the overall certainty of evidence. Secondly, the literature search was limited to studies published in English, which may have introduced language bias. Finally, publication bias cannot be excluded, as studies with negative or less notable findings may be underrepresented in the available literature. These limitations should be considered when interpreting the findings of this review.

## 5. Conclusions

A thorough understanding of the anatomical variations in the STA is essential for surgeons planning free flap reconstruction in the head and neck. This systematic review synthesizes current knowledge regarding STA origin, branching patterns, perfusion territory, and relationship with the SLN. Preoperative imaging, such as CTA, can aid in identifying STA variations and assessing the suitability of the STA as a recipient vessel. Future research should focus on quantifying the impact of STA variations on free flap outcomes and developing standardized reporting methods to facilitate meta-analysis.

## Figures and Tables

**Table 1:** Quality assessment of included studies using AQUA checklist (“+”—present, “-”—missing).

	Title	Abstract	Introduction		Methodology												Results					Discussion				Conclusions	Other Information		
			Background/Rationale	Objective	Study Design and Fundamentals	Setting	Sample Size	Subject	Reference Standard	Outcomes and/or Parameters	Measurement and Assessment	Modality	Technique	Bias	Statistical Approach	Ethics	Subjects	Main Results	Descriptive Anatomy	Confounders	Additional Analyses	Key Findings	Interpretation and Comparison	Implication	Limitation		Acknowledgement	Conflict of Interest	Funding
Abdalla 2024 [1]	+	+	+	+	+	+	+	+	+	+	+	+	+	-	+	+	+	+	+	+	+	+	+	+	+	+	+	-	+
Al-Azzawi 2021 [2]	+	+	+	+	+	-	+	+	+	+	+	+	+	-	+	-	-	+	-	+	-	+	+	+	+	+	+	-	+
Cobiella 2021 [3]	+	+	+	+	+	+	+	+	+	+	+	+	+	-	+	+	-	+	+	+	+	+	+	-	-	+	+	-	-
Estrela 2011 [4]	+	+	+	+	+	-	+	+	+	+	+	+	+	+	+	+	+	+	+	-	+	+	+	-	+	+	-	-	-
Haller 2011 [5]	+	+	+	+	+	-	-	+	+	-	+	+	+	-	+	-	+	+	+	+	-	+	+	-	-	+	-	-	-
Hu 2005 [6]	+	+	+	+	-	-	-	+	+	-	+	+	+	-	-	-	+	+	+	-	-	-	+	-	+	+	+	-	-
Lo 2006 [7]	+	+	+	+	-	+	+	+	+	-	+	+	+	-	+	-	+	+	-	+	+	+	+	+	-	+	+	-	-
Lu 2014 [8]	+	+	+	+	+	-	-	+	+	+	-	+	+	-	-	+	+	+	+	-	-	+	+	+	-	+	+	-	+
Manjappa 2021 [9]	+	+	+	-	+	+	-	+	+	-	+	+	+	-	-	+	-	+	+	-	-	+	+	-	-	+	-	-	-
Ozgur 2009 [10]	+	+	+	+	-	+	+	+	+	+	+	+	+	-	+	+	+	+	+	-	-	+	+	-	-	+	-	-	-
Ozgur 2008 [11]	+	+	+	+	-	+	+	+	+	+	+	+	-	-	+	-	+	+	+	-	-	+	+	-	-	+	-	-	-
Patel 2013 [12]	+	+	-	-	-	+	-	+	+	-	+	-	+	-	-	-	-	+	+	-	-	+	+	-	-	+	+	-	-
Poyraz 2001 [13]	+	+	+	+	-	-	-	+	+	+	+	-	+	-	+	-	+	+	+	-	+	+	+	-	-	+	-	+	-
Al-Rafiah 2011 [14]	+	+	+	+	+	+	-	+	+	+	+	-	+	+	+	-	+	+	+	-	+	+	+	+	-	+	+	-	-
Shyamala 2021 [15]	+	+	+	-	+	+	-	+	+	+	+	+	+	-	-	-	+	+	+	+	-	+	+	-	-	+	-	-	+
Yalcin 2012 [16]	+	+	+	+	+	-	-	+	+	+	-	+	+	-	-	-	-	+	+	+	-	+	+	-	-	+	-	-	-
Yalcin 2014 [17]	+	+	+	+	+	-	-	+	+	+	+	+	+	-	-	-	-	+	+	+	-	+	+	+	-	+	+	-	-
Anand 2021 [18]	+	+	+	+	+	-	+	+	-	+	+	+	+	-	+	-	-	+	+	-	-	+	+	+	-	+	-	-	-
Lucev 2000 [19]	+	+	+	+	+	-	+	+	-	+	+	-	-	-	-	-	+	+	-	-	-	+	+	-	-	+	-	-	-
Bunea 2022 [20]	+	+	+	+	+	+	-	-	+	+	+	+	+	-	-	-	+	+	+	+	-	+	+	-	-	+	-	-	-
Lemaire 2004 [21]	+	+	+	+	+	-	+	+	-	+	+	+	+	+	+	-	-	+	+	+	-	+	+	+	+	+	+	-	-
Lee 2007 [22]	+	+	+	+	+	-	+	+	-	+	+	+	+	-	+	+	+	+	+	-	-	+	+	+	-	+	-	-	-
Kierner 1999 [23]	+	+	+	+	+	+	+	+	-	+	-	+	+	+	-	-	+	+	+	-	-	+	+	+	-	+	+	-	-
Kierner 1998 [24]	+	+	+	+	+	+	+	+	-	+	-	-	+	-	-	-	+	+	+	-	+	+	+	+	-	+	-	-	-
Kapre 2011 [25]	+	+	+	+	+	+	+	+	-	+	-	-	+	-	-	-	-	+	+	-	-	+	+	+	-	+	-	-	-
Gupta 2014 [26]	+	+	+	+	+	+	+	+	-	+	+	+	+	-	-	+	+	+	+	-	+	+	+	+	-	+	-	-	+
Devadas 2016 [27]	+	+	+	+	+	-	+	+	+	-	-	-	+	-	-	-	+	+	+	-	-	+	+	+	-	-	-	-	-
Dessie 2018 [28]	+	+	+	+	+	+	+	+	+	+	+	+	+	-	+	+	+	+	+	-	-	+	+	+	-	+	+	-	-
Wang 1998 [29]	+	+	+	+	+	+	+	-	+	+	+	+	+	+	+	+	+	+	+	+	-	+	+	+	+	+	+	-	-
Devaraja 2021 [30]	+	+	+	+	+	+	+	+	-	+	+	+	+	-	-	+	+	+	+	-	-	+	+	+	+	+	+	-	-
Esen 2017 [31]	+	+	+	+	+	+	+	+	+	+	-	+	+	+	+	+	-	+	+	-	+	+	+	+	+	+	-	+	-
Sharma 2021 [32]	+	+	+	+	+	+	+	+	+	+	-	-	+	-	+	+	+	+	+	-	-	+	+	+	+	+	-	+	-
Salmeron 1998 [33]	+	+	+	+	+	+	+	+	+	+	+	+	+	-	-	-	+	+	+	+	-	+	+	+	+	+	-	-	+
Rusu 2007 [34]	+	+	+	+	+	-	+	+	-	-	-	+	+	-	-	-	+	+	+	+	+	+	+	+	-	-	-	-	-
Rabson 1985 [35]	+	+	+	+	+	-	+	+	-	+	+	+	+	-	-	-	+	+	+	-	-	+	+	+	-	-	-	-	-
Ongeti 2011 [36]	+	+	+	+	+	+	+	+	+	+	-	+	+	-	+	-	-	+	+	-	-	+	+	+	+	+	-	-	-
Park 2013 [37]	+	+	+	+	+	-	+	+	+	+	+	+	+	-	+	+	-	+	+	-	-	+	+	+	+	+	-	+	-
Ahmad 2020 [38]	+	+	+	+	+	+	+	+	+	+	+	-	+	-	+	-	+	+	+	+	+	+	-	+	-	+	+	-	+
Burger 2019 [39]	+	+	+	+	+	+	-	+	+	+	+	+	-	-	-	+	+	+	+	-	+	+	+	+	+	+	+	+	-
Jitpun 2019 [40]	+	+	+	+	+	+	+	+	+	+	+	+	+	-	+	+	+	+	+	-	+	+	+	+	+	+	-	+	-
Griepp 2021 [41]	+	+	+	+	+	+	+	+	+	+	+	+	+	-	+	+	+	+	+	-	-	+	+	+	+	+	+	+	-
Magoma 2012 [42]	+	+	+	-	+	+	+	+	+	+	+	+	+	-	+	-	+	+	+	-	-	+	+	+	+	+	-	-	-
Monfared 2002 [43]	+	+	+	+	+	+	-	+	+	-	+	+	+	-	-	-	+	+	+	-	-	+	+	+	+	+	+	-	-
Herrera Nunez 2020 [44]	+	+	+	+	+	+	+	+	+	+	+	+	+	-	+	+	-	+	+	+	-	+	+	-	+	+	+	+	-
Ortega 2018 [45]	+	+	+	+	+	+	+	+	+	+	+	+	+	+	+	-	+	+	+	-	+	+	+	-	+	+	-	-	+
Sasikumar 2023 [46]	+	+	+	+	+	+	+	+	+	+	+	+	+	-	+	+	+	+	+	-	-	+	+	-	-	+	+	+	+
Sreedharan 2018 [47]	+	+	+	+	+	-	+	+	-	+	+	+	+	-	+	+	+	+	+	-	+	+	-	+	+	-	+	+	+
Kasai 2017 [48]	+	+	+	+	+	+	-	+	+	+	+	+	+	-	+	+	+	+	+	-	-	+	+	+	+	+	-	+	+
Tan 2007 [49]	+	+	+	+	+	+	+	+	-	+	+	+	+	-	-	-	+	+	+	-	-	+	+	-	-	-	-	-	-
Chung 2020 [50]	+	+	+	+	+	+	+	+	+	+	+	-	+	-	+	+	+	+	-	-	-	+	+	+	-	+	-	-	-
Lai 2021 [51]	+	+	+	+	+	+	+	+	+	+	+	-	+	+	+	+	+	+	+	-	+	+	+	+	+	+	+	+	-
Chia 2010 [52]	+	+	+	+	+	+	-	+	-	+	-	-	+	-	-	-	+	+	+	+	+	-	+	+	-	+	-	-	-
Chen 2015 [53]	+	+	+	+	+	+	+	+	-	-	-	+	+	-	+	-	+	+	+	-	+	-	+	+	-	+	+	-	-
Wilson 2012 [54]	+	+	+	+	+	+	+	+	-	+	+	-	+	-	-	-	+	+	+	-	+	+	+	+	+	+	+	+	+

**Table 2:** Origin of the superior thyroid artery [32,47].

Study	ECA (%) [95% Cl]	CB (%) [95% Cl]	CCA (%) [95% Cl]
Sreedharan et al. [47] (*n* = 60)	53 (88.33%) [77.82–94.23]	5 (8.33%) [3.61–18.07]	2 (3.33%) [0.92–11.36]
Sharma et al. [32] (*n* = 30)	27 (90%) [74.38–96.54]	2 (6.67%) [1.85–21.32]	1 (3.33%) [0.59–16.67]
IN TOTAL	80 (88.89%) [80.74–93.85]	7 (7.78%) [3.82–15.19]	3 (3.33%) [1.14–9.35]

Note: 95% CI calculated using the Wilson method. Columns present number of cases, percentage, and confidence interval.

**Table 3:** Influence of gender on STA origin [15,20,32,44].

Author	Nitash Sharma		Maria Christina Bunea		Mario Herrera-Núñez		B.Y. Shyamala [15]	
Gender	Male	Female	Male	Female	Male	Female	Male	Female
External Carotid Artery	90.90%	87.50%	88.33%	86.49%	59%	50%	51%	37%
Carotid Bifurcation	X	X	0%	4.05%	29%	9%	44%	58%
Common Carotid Artery	X	X	11.67%	8.11%	12%	16%	5%	5%

X means that the data was not provided in that research.

**Table 4:** Superior and inferior thyroid arteries anastomoses supplying the parathyroid glands [39].

Parathyroid Glands	Mean Prevalence of STA of ITA Anastomoses	Supply by the Same Vessel on Both Sides
Superior	24.9%	39%
Inferior	6.8%	66%

**Table 5:** Superior thyroid artery supply to sternocleidomastoid muscle [6,23].

Author	Number of Subjects	Area of SCM Supplied by STA
Kierner	31	Middle part
Hu	26	Lower half

**Table 6:** Types of relationships between STA and EBSLN in accordance with Cernea’s classification [28,30,38].

Author	Number of Subjects	1 (95% Cl)	2a (95% Cl)	2b (95% Cl)	No Specific Pattern (95%Cl)
Dessie	43	57% (43.33–71.62)	40.7% (26.37–54.42)	2.3% (0.41–12.06)	-
Ahmad	50	53.2% (40.40–67.03)	17.7% (9.77–30.80)	22.5% (12.75–35.24)	6.4% (0.00–32.44)
Devaraja	8	25% (7.15–59.07)	58.3% (30.57–86.32)	16.6% (2.24–47.09)	-
IN TOTAL	101	52.6% (43.79–62.89)	30.7% (22.54–40.26)	13.4% (7.68–20.78)	3.2% (1.02–8.37)

**Table 7:** Summary of STA, ISLN, ESLN, and ELN as landmarks for use during surgeries [22,37,45].

Structure	Cervical Level	Frequency (%)	Additional Notes
STA	C3–C4	86.7%	Within “safety zone”
STA	C4–C5	26.7%	Partially safe
STA	C5–C6, C6–C7	0%	Unsafe levels
STA	C3	44.4%	Common STA origin level
STA	C3–C4	22.2%	—
ISLN	C3	52.8%	Most common level
ISLN	C2	19.4%	—
ISLN	C3–C4	11.1%	—
ESLN	C3	33.3%	Most common ESLN level
ESLN	C2–C3	13.9%	—
ESLN	C3–C4	22.2%	—
ELN	—	100%	Deep in ascending pharyngeal vein
ELN	—	89%	Passes medial to STA origin
ELN	—	80%	Passes through inferior pharyngeal constrictor
ELN	—	47%	Crosses carotid axis at STA origin

## Data Availability

This study is a systematic review. All analyzed data are available in the original publications cited in the reference list. No new datasets were generated.

## Supplementary Materials

The following supporting information can be downloaded at: https://www.mdpi.com/article/10.3390/jcm14176250/s1; Supplementary File S1: Protocol for Systematic Review: Anatomical Variations of the Superior Thyroid Artery and Implications for Free Flap Surgery; Supplementary File S2: Checklist containing extracted data; Supplementary File S3: AQUA Checklist. Reference [55] is cited in the Supplementary Materials.

## Abbreviations

The following abbreviations are used in this manuscript:

STA	superior thyroid artery
SLN	superior laryngeal nerve
ECA	external carotid artery
CB	carotid bifurcation
CCA	common carotid artery
EBSLN	external branch of superior laryngeal nerve
SLA	superior laryngeal artery
ITA	inferior thyroid artery
SCM	sternocleidomastoid muscle
OMO	omohyoid muscle
THB	tip of the greater horn of the hyoid bone
FA	facial artery
LA	lingual artery
TCA	transverse cervical artery
FFF	free fibula flap
STAP	superior thyroid artery perforator
CTA	computed tomography angiography
