# Supplementary material for: Anatomical Variations in the Superior Thyroid Artery: A Systematic Review and Implications for Free Flap Surgery
Source: J Clin Med. 2025 Sep 5;14(17):6250. doi: 10.3390/jcm14176250 (PMC12429784; doi:10.3390/jcm14176250)
Supplement: Supplementary file 1 [file jcm-14-06250-s001.zip › Supplement S1.pdf]

Protocol for Systematic Review: Anatomical Variations of the Superior Thyroid Artery and Implications for Free Flap Surgery

1. Background and Rationale

The superior thyroid artery (STA) exhibits significant anatomical variability, which has critical implications for head and neck surgical procedures, particularly free flap reconstruction. Understanding these variations is essential for minimizing complications such as hemorrhage, nerve injury, and flap failure. This systematic review aims to synthesize current knowledge on STA anatomy, focusing on its origin, branching patterns, perfusion territory, and relationship with the superior laryngeal nerve (SLN).

2. Objectives

- To systematically review and synthesize the literature on the anatomical variations of the STA.
- To evaluate the implications of these variations for free flap surgery in the head and neck region.
- To provide recommendations for preoperative planning and surgical techniques based on STA anatomy.

3. Eligibility Criteria

Inclusion Criteria:

- **Population:** Human subjects (both living patients and cadavers) of any age, sex, or ethnicity.
- **Setting:** Studies conducted in clinical, surgical, or anatomical laboratory settings.
- **Study Design:** Observational studies, cadaveric studies, imaging studies (e.g., CT angiography), and clinical studies reporting on STA anatomy.
- **Outcomes:** Studies reporting on STA origin, branching patterns, perfusion territory, and its relationship with the SLN (especially the external branch, EBSLN).
- **Language:** English-language studies.

Exclusion Criteria:

- **Animal Studies:** Studies conducted on non-human subjects.
- **Fetal Studies:** Studies conducted on fetuses.
- **Non-Anatomical Studies:** Studies that do not provide detailed anatomical data on the STA.

- **Case Reports:** Single case reports or small case series with fewer than 5 subjects, unless they provide unique anatomical insights.
- **Non-English Publications:** Studies published in languages other than English.
- **Insufficient Data:** Studies that do not provide sufficient detail on STA anatomy or its variations.

4. Search Strategy

Databases to be Searched:

- PubMed
- Embase
- Scopus
- Web of Science
- EBSCO
- Google Scholar

Search Terms:

- Superior thyroid artery anatomy
- Superior thyroid artery surgery

5. Study Selection Process

1. **Initial Screening:** Titles and abstracts of identified studies will be screened for relevance based on inclusion and exclusion criteria.
2. **Full-Text Review:** Potentially relevant studies will undergo full-text review to determine eligibility.
3. **Data Extraction:** Data from included studies will be extracted using a standardized form, including study design, population, anatomical findings, and surgical implications.
4. **Discrepancies:** Any discrepancies in study selection or data extraction will be resolved through discussion between reviewers or consultation with a third reviewer if necessary.

6. Data Extraction

Data to be Extracted:

- Study characteristics (author, year, aim of study, type of study, methodology, sample size).

- STA origin (external carotid artery, carotid bifurcation, common carotid artery).
- STA branching patterns (number and type of branches).
- STA perfusion territory (thyroid gland, larynx, sternocleidomastoid muscle, strap muscles).
- Relationship between STA and SLN (Cernea classification, distances, risk of injury).
- Implications for free flap surgery (suitability as a recipient vessel, surgical landmarks).

7. Risk of Bias Assessment

The risk of bias in included studies will be assessed using appropriate tools:

- **For anatomical studies:** The Anatomical Quality Assessment (AQUA) tool.

8. Data Synthesis

- **Qualitative Synthesis:** Findings will be summarized narratively, with a focus on common anatomical variations and their surgical implications.

9. Subgroup Analysis

If data allow, subgroup analyses will be performed based on:

- **Sex:** Male vs. female.
- **Laterality:** Right vs. left STA.
- **Geographical Region:** Variations across different populations.

10. Reporting

The review will be reported according to the **PRISMA (Preferred Reporting Items for Systematic Reviews and Meta-Analyses)** guidelines. A PRISMA flow diagram will be included to illustrate the study selection process.

11. Timeline

- **Literature Search:** Month 1
- **Study Selection and Data Extraction:** Month 2
- **Risk of Bias Assessment:** Month 3
- **Data Synthesis and Analysis:** Month 3-4
- **Manuscript Preparation:** Month 5-6
- **Submission for Publication:** Month 6

12. Ethical Considerations

Since this is a systematic review of existing literature, ethical approval is not required. However, all data will be handled confidentially, and proper attribution will be given to original authors.
